# Chicago Medical Society

**Published:** 1882-07

**Authors:** 


### Society Reports.

### ARTICLE XII.

### CHICAGO MEDICAL SOCIETY.

Stated Meeting, July 3, 1882. Dr. J. H. Hollister, President, in the chair.

After reading of the minutes of the last meeting by Dr. Liston H. Montgomery, a motion was passed that hereafter honorary members of this society who desired becoming active members, would be so regarded, provided they were resident physicians and paid the annual dues.

Discussion on the causes of, and treatment for preventing the mortality in children, from serous diarrhœa and cholera infantum.

Dr. Fenn opened the discussion, which had been postponed at the last meeting, at which Dr. N. S. Davis had read a paper on this important subject. Dr. Fenn believed that there was no special noxious agent causing this disease; that, in his opinion, the same influences that affected adults affected children likewise. Everybody knew that heat was a great stimulant, which, unduly increased, was destructive in its effects. The susceptibilities of children were greater than those of adults, and under the steady influence of heat their vitality grew less. There were many trivial causes of diarrhœa in children. In our northern latitude, the labors of the day enable adults to rest well in the night, but children were often restless and could not sleep. Sometimes they lay uncovered, and while their side next to the bed was sweating, the one exposed to the air was chilled, and thus the taking of cold was a frequent cause of diarrhœa. Another cause was the constant hugging of children by their mothers. They were kept as if wrapped in a poultice; yet, the exhalations from adult bodies were deleterious to infants. There was a necessity on the part of parents to change their children's clothes frequently, and bathe them in cold water. Fresh air, good food and pure water were indispensable in the treatment of the disease under consideration. Sometimes abstinence from all food except milk and lime-water was necessary. But he deprecated the use of powerful or debilitating medicines.

Dr. D. M. Tucker used calomel and soda in gastro-enteritis, with bismuth and syr. rhubarb whenever indicated. He advised removal of the child and mother to the country when practicable, for the city was a too fertile soil for the disease to feed on. He would give children no solid food until they had teeth to masticate it. The mother should nurse the infant by all means. Warm applications, 70 F., should be made to the abdomen, and if there was a catarrhal affection of the lungs, a frequent concomitant, that should be attended to.

Dr. Paoli said that catarrhal gastro-enteritis and cholera infantum were fatal diseases in all the great cities; that remedies would fail in spite of all ability on the part of physicians, even after the best hygienic precautions had been taken. But many physicians called cholera infantum a mere congestion of the bowels, and got the credit of a speedy cure. As to treatment, we had to individualize it and adapt it to the case as might be. There were gastritis, colitis and enteritis, each of which might assume various forms. There were the poor, who lived in the midst of hurtful surroundings, which could not be ameliorated, and where the mothers gave indigestible food to their children, in spite of the physician's advice. Then grave complications, as dysentery, sometimes set in. As a valuable remedy, he recommended small doses of nitrate of silver, 1–20 gr. every three hours.

Dr. S. Strausser said that a most frequent cause of the diarrhœa of children among German populations was prolonged lactation. In his opinion, a child eight months old should be weaned if there was a tendency to diarrhœa, and lactation should never be prolonged after the twelfth month. He had met several cases in which the cause of the disease was phymosis, and circumcision cured them. In all these cases, the closest attention should be given to the diet. He enjoined perfect rest; abstinence from food in many cases; a flaxseed poultice with a little mustard in it, to be applied to the belly, and a warm bath, in some cases. He gave bismuth subnitrate with the chalk mixture, and paregoric if indicated. In vomiting, he gave aromatic spirits of ammonium. He believed that under the treatment which he had just indicated the mortality would be reduced one-half. Infant food was worse than starvation. Milk with lime-water, and a few drops of whisky in ice-water, were good drinks in such cases.

In gastro-enteritis, Dr. J. H. Bates believed that the first symptoms were those of irritation of the nervous system, be the cause what it may, heat or indigestion. In such cases, care should be taken, when giving cows' milk, that it came from healthy cows. It was advisable to clothe the children in wool. Some cases of diarrhœa were malarial, recurred in periodical aggravations, and were best treated with quinine, a remedy which sometimes produced its effect on the infant through the milk, if administered to the nurse. But he did not believe in the efficiency of any medicine in particular.

Dr. Earle said that cholera infantum was the rarest of diseases. Although he met a great many cases of diseases of children, he seldom came across a case in the space of a year; but simple diarrhœa, entero-colitis and dysentery were quite common. The two first of these were often the result of bad diet. One of the remedies he prescribed the most was phosphate of sodium. He gave as much as would hold on a three cent piece at a dose. This was the salt found in human milk; in which it differed from that of any other animal. He did not advise weaning a child sick with diarrhœa. If extra food was required, he gave rice-water, with just enough cream to make it nutrient, and seasoned with sugar and salt. He withdrew milk in cases in which he found that it caused the disease, and did the same in regard to other diets, as the case might be. We had sometimes to avail ourselves of artificial foods. He had observed that comdensed milk was given in too small quantities. Children were starved on it. Some children never could satiate their appetite, and had never been allowed a good square meal. He gave one teaspoonful of condensed milk, with seven of rice-water, at a time.

For twenty-five years, Dr. J. H. Hollister had given the subject a good deal of thought. He believed that an atmospheric temperature of at least 70° F., continued for a few days, was required for that form of the disease known as summer complaint to settle in the community. He explained the occurrence thus: A prolonged high temperature caused paresis of the nervous ganglia, and through the action of the vaso-motor nerves, the vessels of the secretory organs were relaxed. Functional derangement of the liver arose, and chills took place. A serous discharge from the bowels was the inevitable result of congestion.

As to treatment, the temperature should be brought down, in acute cases, by cooling and bathing. He believed that calomel was a nervous stimulant, as the salts of zinc; that it acted on the ganglia of the nervous system, and relieved the congestion of the bowels. For twenty years he had relied on it in such cases. In diarrhœas, after inflammation had set in, the most favorable stage was passed, and these cases were often looked upon by mothers as results from dentition which should not be interfered with. In his practice, the doctor often tested the discharges with litmus paper, and then administered alkalies, if they were acid. He had used calomel, in the dose of $\frac{1}{4}$ to $\frac{1}{8}$ of a grain, as a nerve-stimulant, with good results. When there was excessive irritability of the mucous membrane, he gave the camphorated tr. of opium one to five drops; also the chalk mixture.

℞ Tr. opii camphor,<br>

Mist. cretæ, } ........................āā ℨi.<br>

Sacchari.<br>

Aquæ........................................ad ℥ij.

M. Sig. teaspoonful every three hours for a child more than a year old.

He had been very well satisfied with this *positive* treatment, and had lost few cases. The most harmful circumstance was that mothers erroneously believed that teething diarrhœa should not be checked. In extreme prostration he gave a few drops of brandy.

Dr. Lester Curtis, after experimenting, had come to regard calomel as an efficient remedy in much smaller doses than usually given. He divided $\frac{1}{3}$ gr. of the remedy into twelve powders with sugar, and gave it every three hours. He had obtained beneficial results from as low as one-sixtieth of a grain.

The society adjourned.

### PATHOLOGICAL SOCIETY.

At the meeting of the Pathological Society held June 26, after Dr. Lydston's paper on the treatment of erysipelas, an animated discussion followed. Dr. E. W. Lee recommended the use of alcoholics internally, and carbolic acid locally. In the cutaneous form, he gave one part of it to twenty of a mixture of gum tragacanth, acacia and molasses. He also recommended the local use of steamed compresses. Dr. Odelia Blinn recommended the external use of chloroform in rebellious cases.

Dr. D. R. Brower had obtained very beneficial results from the use of belladonna internally. It was established by Ringer and by Bartholow, and deserved a better trial than it had received yet.

H. D. V.

---

AS a profession, doctors think it undignified to have much to do with politics. As a whole, they are not as big tax-payers as they ought to be, but numerically strong, intelligent and influential. Unfortunately, other of the better elements of society hold themselves aloof from mingling in what they term the filthy pool of politics. This condition ought not to be, if we want to break down bossism and machine politics, to defeat vicious and secure good legislation. 'Twould be a misfortune for all doctors to think alike, but if each will follow his convictions into the ranks of that politcal party nearest his liking, then exert his influence toward sending intelligent, representatative men to the legislature, we may secure the enactment of such laws as will protect the people, and convince them that we have been impelled by motives other than a desire to attain selfish ends.—*Dr. Crumpton*, in *Pacific Med. and Surg. Journal.*

